# Food additives with aldehyde skeleton exhibited leptin resistance in cells

**DOI:** 10.3389/fnut.2026.1892425

**Published:** 2026-07-23

**Authors:** Yuki Shibuya, Haruto Yoshida, Kyoshiro Tsuge, Yu Hamasaki, Karen Kuriya, Tadashi Nakagawa, Aoi Sugiyama, Kazuhide Miyamoto, Akira Shimamoto, Ralf Jockers, Julie Dam, Toru Hosoi

**Affiliations:** 1 Department of Pharmacotherapy, Graduate School of Pharmaceutical Sciences, Sanyo Onoda City University, Sanyo Onoda, Yamaguchi, Japan; 2 Department of Regenerative Medicine, Graduate School of Pharmaceutical Sciences, Sanyo Onoda City University, Sanyo Onoda, Yamaguchi, Japan; 3 Department of Biophysical Chemistry, Graduate School of Pharmaceutical Sciences, Sanyo Onoda City University, Sanyo Onoda, Yamaguchi, Japan; 4 Université Paris Cité, Institut Cochin, INSERM, CNRS, Paris, France

**Keywords:** endoplasmic reticulum stress, food additives, leptin resistance, obesity, signal transducer and activator of transcription 3

## Abstract

Obesity, a major health issue linked to metabolic disorders, is driven by multiple factors, including impaired leptin signaling, particularly in the Janus kinase 2 (JAK2)/Signal transducer and activator of transcription 3 (STAT3) pathway. This study investigated the impact of aldehyde-containing food additives on leptin receptor activated signaling in cells. We examined the effects of 39 different aldehyde-containing compounds, commonly used in food additives, on leptin-induced STAT3 transcriptional activity through a reporter gene assay. Three aldehydes, i.e., 2-pyrrolecarboxaldehyde, o-anisaldehyde, and piperonal were found to reduce leptin-induced STAT3 transcriptional activity in a concentration-dependent manner without affecting cell viability. These additives did not alter conformational changes of the leptin receptor, STAT3 phosphorylation, or its nuclear translocation, nor did they induce endoplasmic reticulum stress. These additives likely affect a downstream step in leptin signaling, specifically transcriptional regulation by STAT3, without disrupting early receptor signaling events. These finding suggest that some aldehyde-based food additives may contribute to leptin resistance and potentially to the development of obesity.

## Introduction

Obesity-related pathologies include metabolic syndrome, type 2 diabetes, hypertension, dyslipidemia and cardiovascular diseases, and have become a major public health concern. The World Obesity Federation (WOF) has projected that by 2035 more than half of the global population will be overweight or have obesity, with an estimated economic burden of USD 4.32 trillion (From WOF homepage: https://www.worldobesity.org/). It is important to reduce obesity rate for reducing non-communicable diseases (NCD) (1). According to the report from the World Health Organization, 75% of non-pandemic-related deaths are due to NCD (2). Both environmental and genetic factors contribute to the development of obesity-related conditions (3). However, despite a growing understanding of the risks of obesity, the precise mechanisms underlying its pathogenesis remain unclear. One of the possible environmental factors that may affect overweight is ultra-processed foods (UPF) (4, 5). Epidemiological study suggested that consuming UPF causes increased calorie intake and weight gain (6, 7). Also, various type of food additives contained in UPF were suggested to contribute to obesity (8). However, how exposure of these food additives contribute to obesity remain unknown.

Leptin is a widely recognized anti-obesity hormone (9). Leptin is mainly secreted from adipose tissue, and the circulating leptin will enter into the hypothalamus (10, 11). In the hypothalamus, leptin will majorly act on neuronal cells of leptin receptor and activates JAK2/STAT3 signaling (12, 13). By activating neuronal cells, leptin inhibits appetite and increases energy expeditor, all of which will decrease body weight. Adequate fat stores and leptin concentrations promote neuroendocrine and autonomic-driven energy expenditure, while reducing food intake. Clinically, circulating leptin is commonly used as a biomarker for obesity. However, patients with obesity treated with leptin show no improvement of their condition (14–16). In a state of such a condition, leptin’s action in inducing JAK2/STAT3 signaling were impaired (17, 18). Nowadays, such a condition, i.e., leptin resistance, is suggested to play key role in the development of obesity (19). This resistance to leptin is often compared to the concept of insulin resistance (20). While reduced leptin responsiveness contribute to obesity, the molecular mechanisms underlying leptin resistance are not well understood. Understanding the mechanisms of leptin resistance is important with respect understanding the mechanisms of obesity as well as depression and type 1 diabetes, as the leptin is reported to be involved in these diseases (21–23). Considering the development of leptin resistance, endoplasmic reticulum (ER) stress is suggested to be one of the underlying mechanisms (24, 25).

ER stress refers to a condition where ER homeostasis is disrupted, resulting in the accumulation of misfolded proteins. The ER plays a crucial role in the regulation of protein homeostasis; and when cells perceive stress stimuli that disrupt ER function, unfolded proteins accumulate in the ER, causing ER stress (24, 26). Three major branches of the unfolded protein response (UPR) pathway have been reported: the double-stranded RNA-activated protein kinase-like ER kinase - eukaryotic initiation factor 2α (eIF2α), the inositol-requiring enzyme-1-X-box binding protein 1 (XBP-1) and the activating transcription factor 6 pathway (27–30). We and others have previously reported that ER stress may contribute to the development of leptin resistance (31, 32). Recently, molecular mechanisms of the ER stress-induced leptin resistance were reported (33, 34), and pharmacological strategy for obesity targeting ER stress has been proposed (35, 36).

We have previously shown that the biological aldehyde 4-hydroxynonenal (4-HNE), induces ER stress response and contributes leptin resistance (37). 4-HNE is typically generated when reactive oxygen species (ROS) damage cell membrane lipids (38). These findings suggest that aldehyde-containing chemical products compounds may affect leptin signaling. However, it is currently unknown whether food additives containing aldehyde skeleton could also affect leptin signaling.

In the present study, we screened 39 different aldehydes found in food additives to assess their potential to induce leptin resistance in cellular models. Currently, we investigated whether food additives attenuate leptin-induced JAK2/STAT3 signaling.

## Results

### Aldehyde-containing food additives suppress leptin-induced STAT3 reporter gene activity

**Figure 1:**
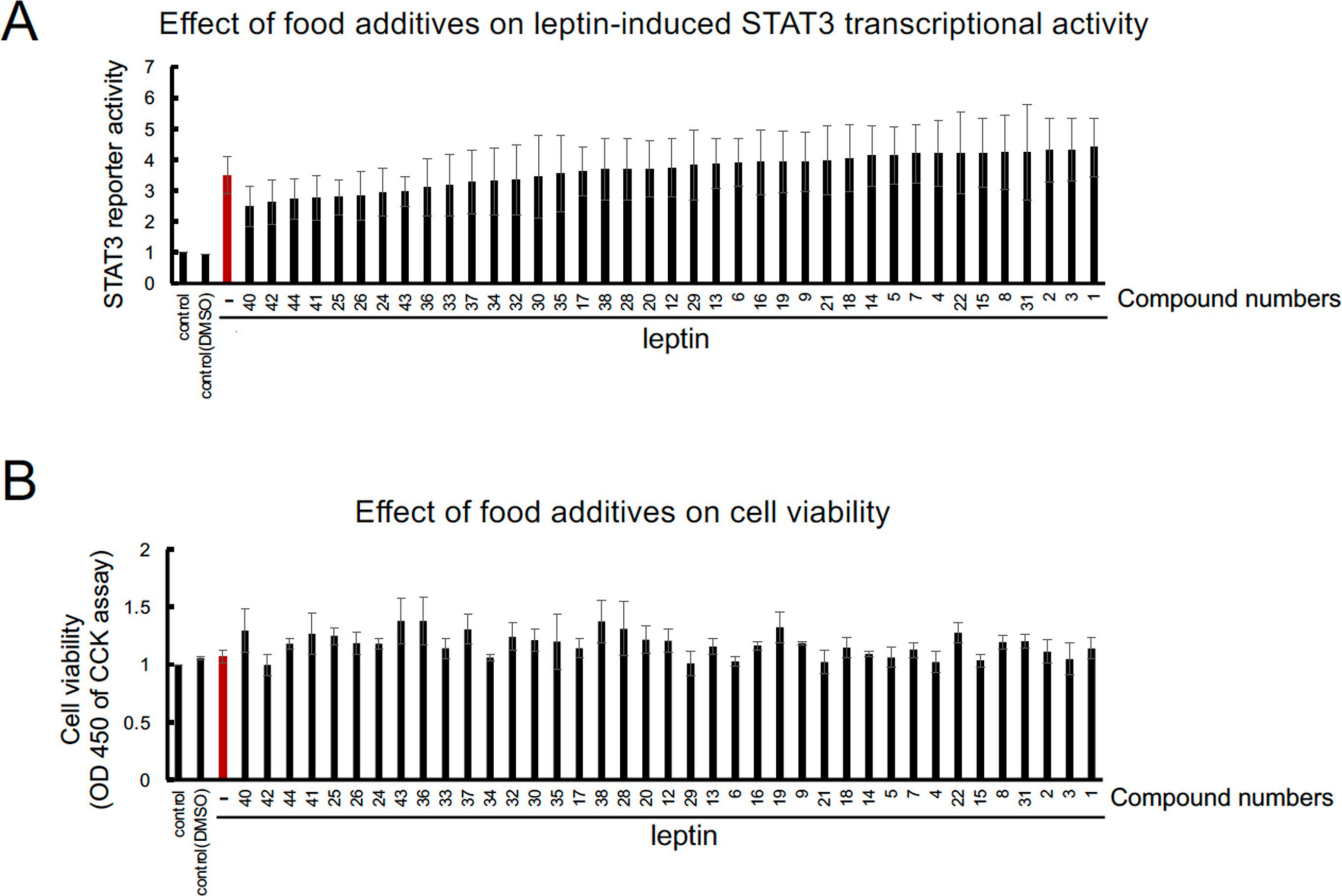
Identification of food additives, which inhibits leptin-induced transcriptional activity SH-SY5Y neuroblastoma cells expressing Ob-Rb (SH-SY5Y-Ob-Rb SIE-luc2P) were treated with the indicated aldehydes (30 μM) for 3 h followed by addition of leptin (0.01 μg/mL) for the last 2 h. **(A)** Treatment with leptin alone increased STAT3 transcriptional activity (red bar of **A**). We then analyzed the effect of various compounds on leptin-induced signaling (black bar of **A**). The level of the STAT3 activity was analyzed by luciferase assay. **(B)** The cell viability after treatment with the compounds was analyzed by CCK assay. These compounds did not affect cell viability. Values are presented as means ± S. E. *n* = 4.

**Figure 2:**
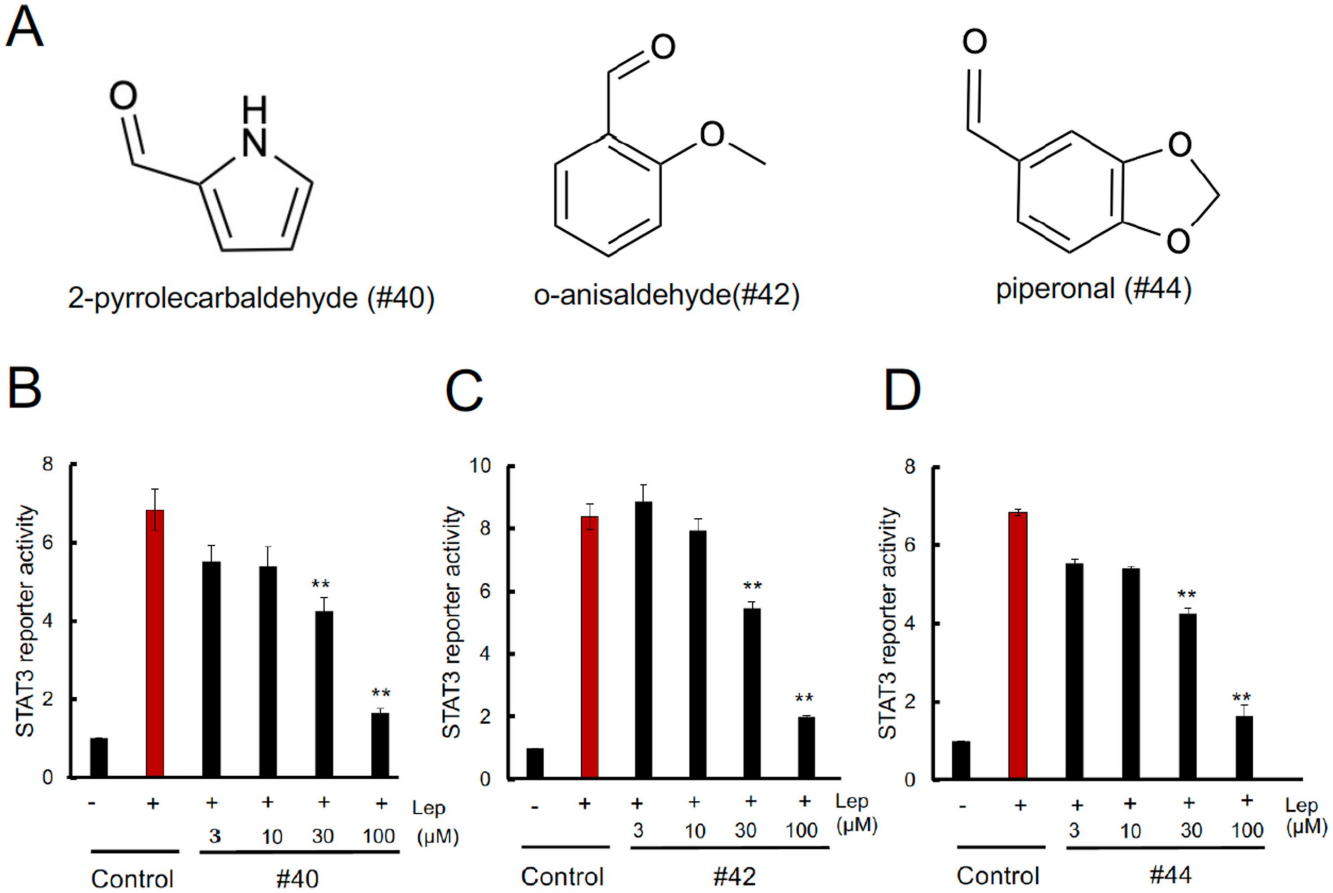
Concentration-dependent effect of food additives (#40, 42, 44) on leptin-induced transcriptional activity. Leptin alone increased STAT3 transcriptional activity (red bars in **B,C,D**). Food additives #40, 42, 44 (2-pyrrolecarbaldehyde, o-anisaldehyde and piperonal) inhibited leptin-induced STAT3 transcriptional activity in a concentration dependent manner (black bars in **B,C,D**). **(A)** Effect of 2-pyrrolecarbaldehyde (#40, 3–100 μM) on leptin (0.01 μg/mL, 3 h)-induced STAT3 transcriptional activity. Values are presented as means ± S. E. **p < 0.01 vs. control cells treated with leptin. *n* = 3, Tukey–Kramer and Dunnett’s test. Leptin v.s. leptin + #40 (100 μM): d = 7.89 at Cohen’s d test **(B)** Effect of o-anisaldehyde (#42, 3–100 μM) on leptin (0.01 μg/mL, 3 h)-induced STAT3 transcriptional activity. Data are presented as mean ± S. E. **p < 0.01, *p < 0.05, *n* = 4. leptin v.s. leptin + #42 (100 μM): d = 11.21 at Cohen’s d test **(C)** Effect of piperonal (#44, 3–100 μM) on leptin (0.01 μg/mL, 3 h)-induced STAT3 transcriptional activity. Values are presented as means ± S. E. **p < 0.01 vs. control cells treated with leptin. *n* = 4. leptin v.s. leptin + #44 (100 μM): d = 12.42 at Cohen’s d test.

First, we investigated whether food additives containing aldehyde structures affect leptin-induced JAK2-STAT3 signaling pathway. To this end, we conducted a screening assay using SH-SY5Y-ObRb cells expressing the sis-inducible element (SIE) linked to the luciferase reporter gene, to monitor the activation of the JAK2-STAT3 pathway. Thirty-nine aldehyde-containing compounds, which are used as food additives, were randomly selected, and added to cells 1 h before leptin addition for an additional 2 h. STAT3 transcriptional activity and cell viability was subsequently analyzed. The screening results revealed that several compounds attenuated leptin-induced STAT3 transcriptional activity without affecting cell viability (Figures 1A,B). To confirm the inhibitory effect of these compounds, concentration-dependent effects were examined. Three compounds, i.e., #40: 2-pyrrolecarbaldehyde, #42: o-anisaldehyde and #44: piperonal, were selected for dose–response analysis (3, 10, 30, 100 μM) on leptin-induced STAT3 transcriptional activity. A concentration-dependent attenuation was observed with significant reductions at 30 and 100 μM (Figure 2). These findings indicate that certain aldehyde-containing compounds may inhibit leptin-induced STAT3 transcriptional activity.

### The inhibitory effect of aldehyde-containing compounds on leptin signaling does not involve the ER stress

**Figure 3:**
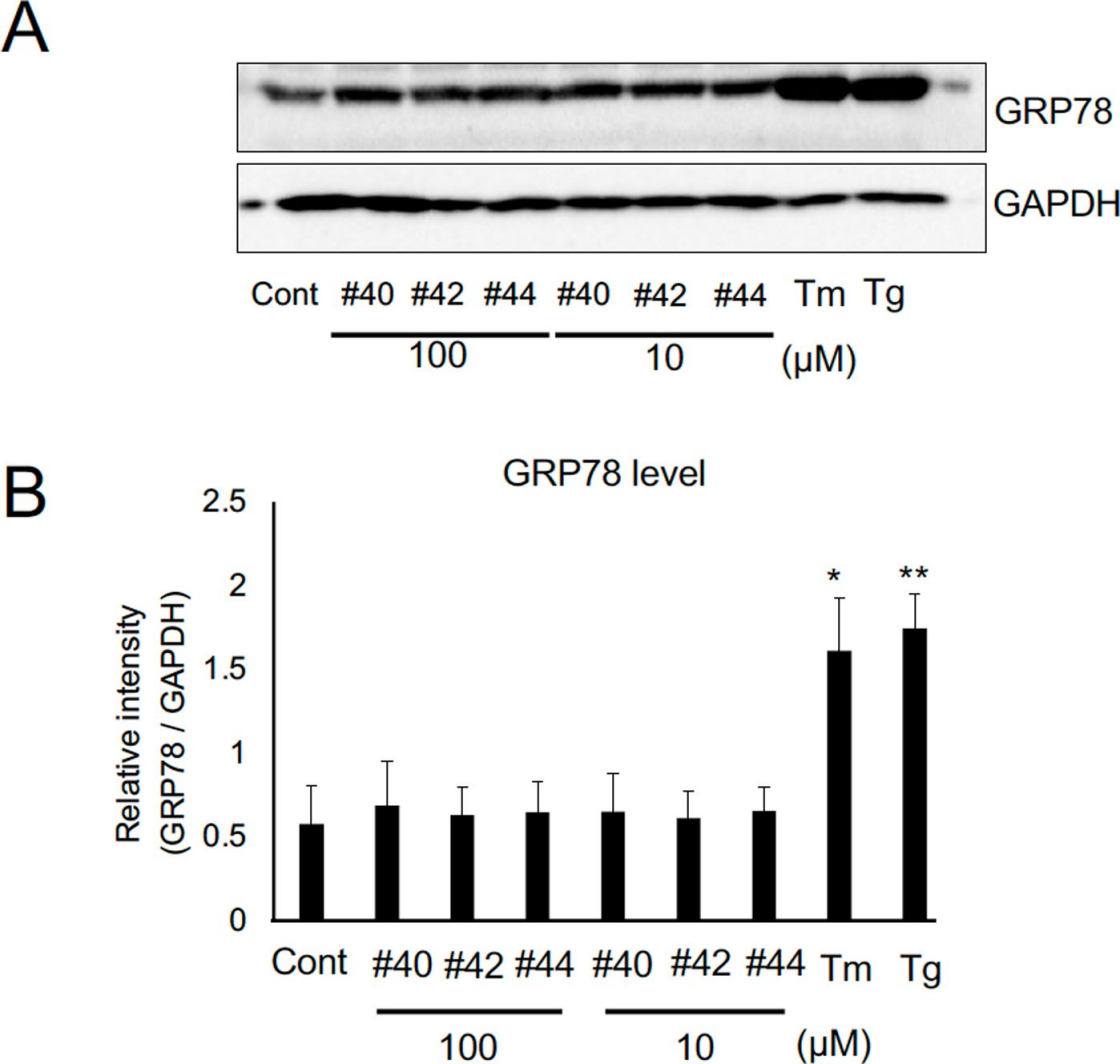
Food additives (#40, #42, #44) did not induce ER stress pathway. The cells were treated with food additives (#40, #42, #44), and analyzed GRP78 induction as an ER stress indicator. Food additives (#40, #42, #44) did not induce GRP78 induction. As a positive control, treatment with Tm and Tg increased GRP78 induction. **(A)** 2-pyrrolecarbaldehyde (#40), o-anisaldehyde (#42), piperonal (#44) were treated for 16 h at 100 μM and Western blotting were performed. **(B)** Quantification of Western blot results, normalized by GAPDH. Values are presented as means ± S. E. **p < 0.01 vs. control cells treated with leptin. *n* = 3. cont v.s. Tm: d = 3.55 at Cohen’s d test, cont v.s. Tg: d = 2.77 at Cohen’s d test.

We previously reported that ER stress inhibits leptin signaling (31, 35). To further investigate the specific molecular mechanisms of food additives, we examined the impact of compounds #40, #42, #44 on the ER stress pathway by monitoring induction of glucose regulated protein 78 (GRP78) expression in SH-SY5Y-ObRb cells. Whereas GRP78 was readily observed upon treatment with two well-established ER stress-inducing reagents, tunicamycin (Tm) and thapsigargin (Tg), none of the aldehyde-containing compounds (at 10, 100 μM, 16 h) had effect (Figure 3). These results indicate that compounds #40, #42, and #44 are not likely to induce ER stress, and that the inhibitory effect of aldehyde-containing compounds on leptin signaling does not involve the ER stress pathway.

### Aldehyde-containing compounds inhibit leptin signaling without affecting leptin receptor activation

**Figure 4:**
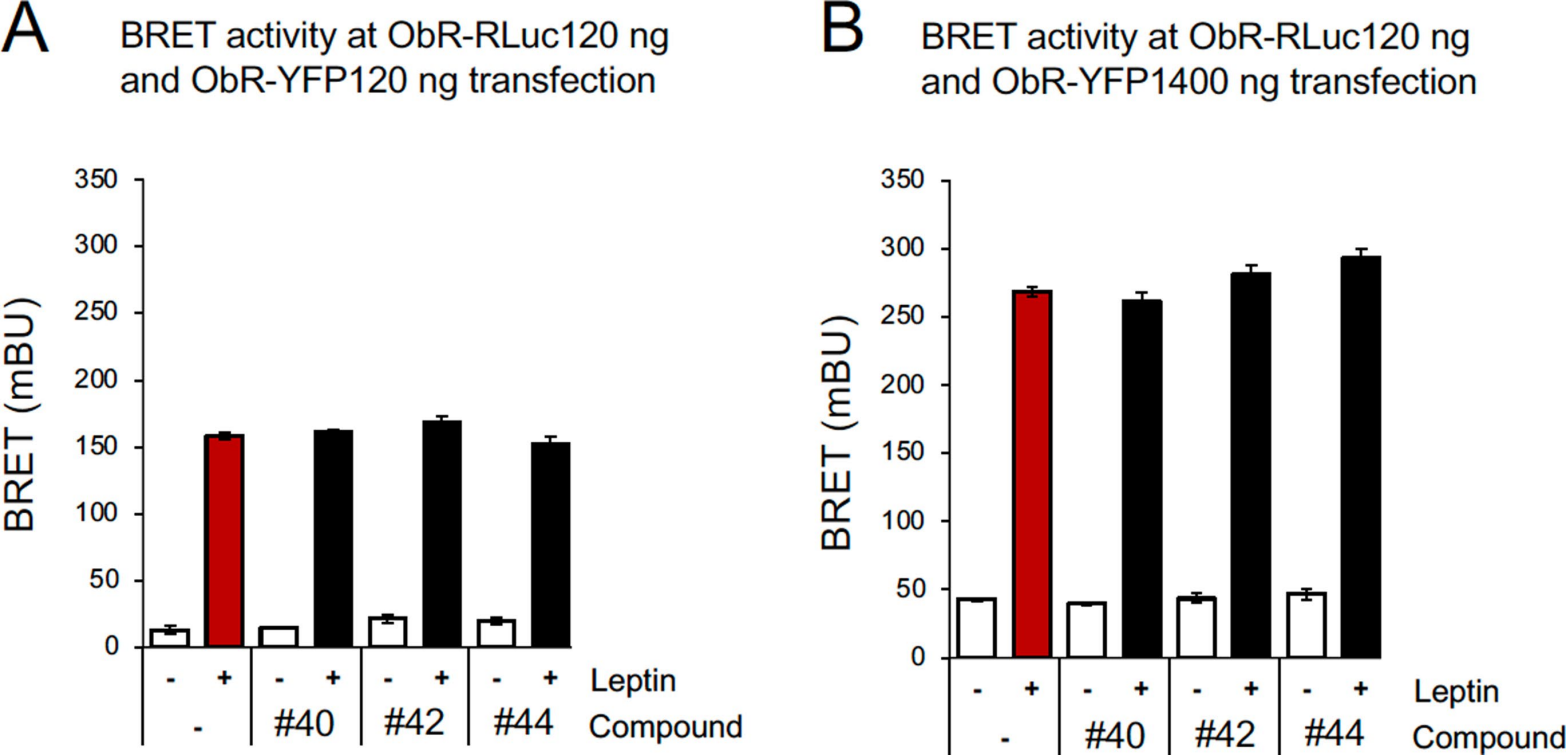
Food additives (#40, #42, #44) did not affect leptin-induced molecular rearrangements within the Leptin Receptor complex. HEK293T cells transfected with the leptin receptors, ObR-Rluc and ObR-YFP, were treated with the food additives and analyzed leptin-induced conformational changes of leptin receptor by BRET analysis. Leptin (10 nM, 30 min) increased BRET signal, indicative of conformational changes of leptin receptor. However, food additives (#40, #42, #44) did not affect leptin (10 nM, 30 min)-induced conformational changes of leptin receptor. **(A)** Results of the BRET transfected with ObR-Rluc (120 ng) and ObR-YFP (120 ng). **(B)** Results of the BRET transfected with ObR-Rluc (120 ng) and ObR-YFP (1,400 ng).

We next examined a possible effect of the compounds directly on leptin receptor activation by monitoring the leptin-induced rearrangement of Ob-R complex using Bioluminescence Resonance Energy Transfer (BRET) biosensors. As expected, leptin induced a robust BRET signal in HEK293T cells, indicative of conformational changes between the two BRET pairs, OBR-Rluc protomer and OBR-YFP protomer, at the plasma membrane as expected (39) (Figure 4). Pretreatment of cells with compounds #40, #42, and #44 (100 μM, 1 h) did not modify the leptin response of the BRET pairs expressed at two different ratios, ratios close to BRET50 and at BRETmax (Figures 4A,B). BRET50 corresponds to the concentration at which half of the maximum BRET signal is achieved, while BRETmax represents the maximal BRET signal, indicating the full extent of receptor activation. These results indicate that the compounds do not directly affect Ob-R activation itself.

### Reduced leptin-regulated STAT3 activity is not due to altered STAT3 phosphorylation or nuclear translocation

**Figure 5:**
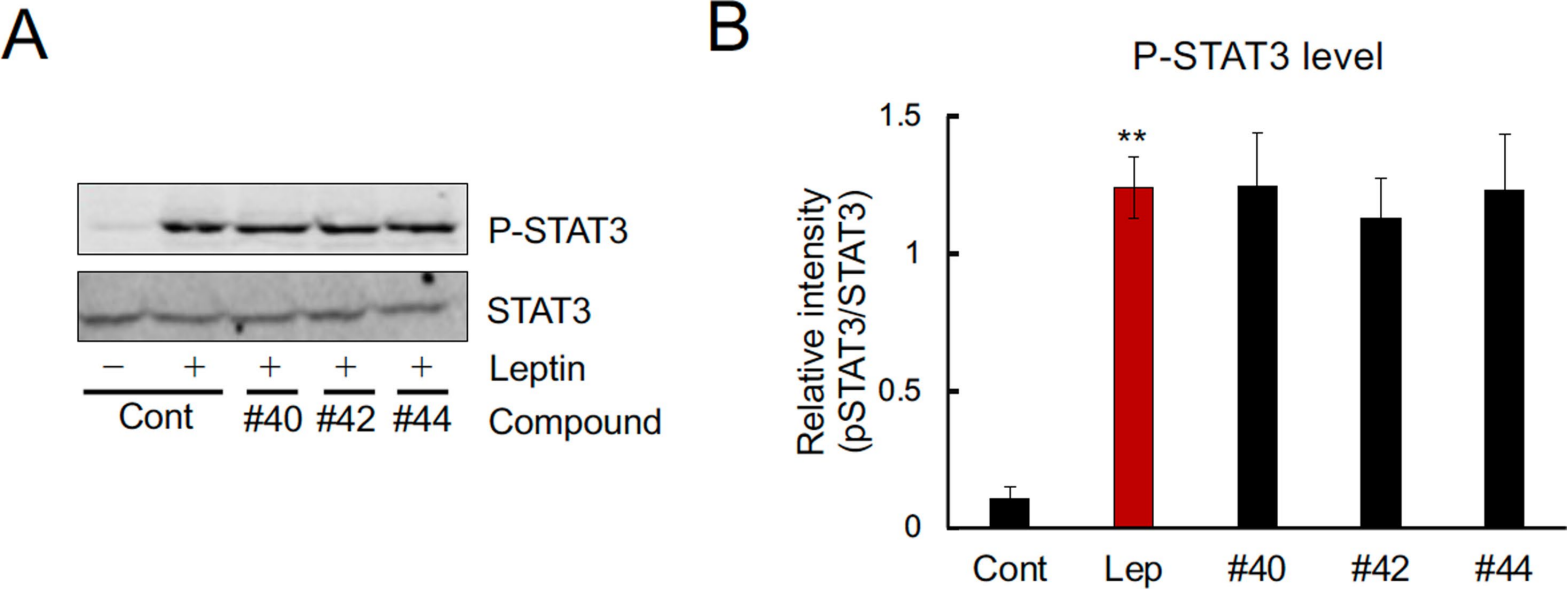
Food additives (#40, #42, #44) did not affect leptin-induced STAT3 phosphorylation. The effect of food additives (#40, #42, #44) on leptin-induced STAT3 phosphorylation was analyzed. Leptin induced STAT3 phosphorylation (red bar of **B**). However, food additives (#40, #42, #44) did not affect leptin-induced STAT3 phosphorylation (black bar of **B**). **(A)** SH-SY5Y-Ob-Rb cells were pretreated with food additives (#40, #42, #44) and leptin-induced STAT3 phosphorylation (p-STAT3) was analyzed by Western blotting. Food additives (#40, #42, #44) did not affect phosphorylation levels of STAT3 under the leptin stimulation. **(B)** Quantification results of Western blot analysis. Values are presented as means ± S. E. **p < 0.01 vs. control cells treated with leptin. *n* = 5. cont v.s. leptin: d = 5.65 at Cohen’s d test.

**Figure 6:**
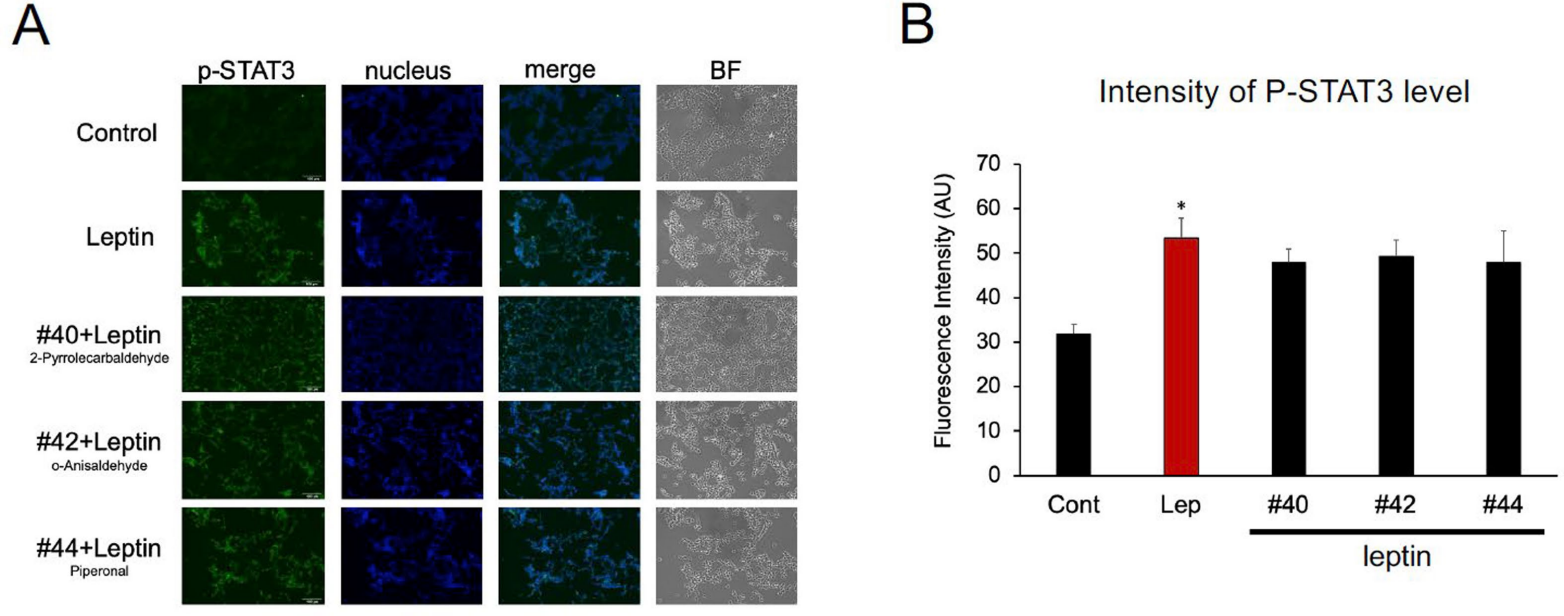
Food additives (#40, #42, #44) did not affect leptin-induced nuclear translocation of phospho-STAT3. Leptin induced nuclear induction of phospho-STAT3 (red bar of **B**). Food additives (#40, #42, #44) did not affect the nuclear induction of phospho-STAT3 (black bar of **B**). **(A)** Representative immunofluorescence images of phospho-STAT3 (p-STAT3) in cells treated with leptin and food additives (#40: 2-pyrrolecarbaldehyde, #42: o-Anisaldehyde, #44: piperonal) observed using optical microscope. Addition of the food additives on leptin induced phospho-STAT3 levels were visualized by immunofluorescence staining. **(B)** Quantitative analysis of phospho-STAT3 immunofluorescence intensity. Leptin-induced phospho-STAT3 levels were quantified using the mean fluorescence intensity (green) of phospho-STAT3 as a measure. Analysis revealed a significant increase in phospho-STAT3 levels compared to the control group. Data are presented as means ± S. E., *n* = 4. *; *p* < 0.05 vs. control group. Cont v.s. leptin: d = 3.06 at Cohen’s d test.

We next evaluated the effect of the compounds on leptin-induced STAT3 phosphorylation, a direct downstream consequence of Ob-R activation. Stimulation of SH-SY5Y-Ob-Rb cells with leptin showed the expected increase in the phosphorylation of STAT3 as monitored by WB (Figure 5A). The presence of food additives (#40, #42, and #44), did not modify the leptin-induced phosphorylation of STAT3 (Figure 5B). We next explored whether the food additives may affect the nuclear translocation of Phospho-STAT3 (40). Treatment with leptin induced the expected increase in nuclear Phospho-STAT3 in SH-SY5Y-Ob-Rb cells as monitored by immunostaining which was not affected by the co-treatment with food additives i.e.; #40, #42, and #44 (100 μM; Figures 6A,B). These results suggest that the food additives do not affect leptin-induced STAT3 phosphorylation and nuclear translocation of phospho-STAT3.

## Discussion

In the present study, we investigated the potential involvement of food additives containing aldehyde structures in the etiology of leptin resistance and explored their potential molecular mechanisms. Our findings revealed that three types of food additives with aldehyde structures decrease the activity of the STAT3 reporter gene in leptin-activated cells.

Given that ER stress is a well-established mechanism in the development of leptin resistance (13, 31, 32, 41–43), and that an aldehyde-based compound, 4-hydroxy-2-nonenal, was shown to induce ER stress (37), we hypothesized that aldehyde-containing compounds in certain food additives could induce ER stress, thereby contributing to leptin resistance. However, our results showed that three aldehyde compounds (#40, #42, #44) did not induce a significant ER response, as assessed by GRP78 induction (Figure 3). This observation suggests that, contrary to our hypothesis, ER stress is not the mechanism through which these food additives impair leptin response. These findings pave the way for exploring other potential mechanisms through which these additives might interfere with leptin signaling. Importantly, the compounds did not affect key upstream parameters such as receptor activation, STAT3 phosphorylation, or STAT3 nuclear translocation, regulated by leptin. At this stage, the precise mechanism of action remains unclear, but these additives likely influence a downstream step in leptin signaling, potentially through the transcriptional regulation by STAT3, rather than disrupting early events at the receptor level or within the signaling pathways. This is consistent with studies showing that other food additives, such as dietary emulsifiers, can alter gene expression in the mouse brain (44), and that common additives like carrageenan can inhibit proglucagon expression and GLP-1 secretion in human enteroendocrine L-cells (45). As well as STAT3 signaling, leptin can also activate mitogen-activated protein kinases (MAPK) and PI3K/AKT signaling pathways (46, 47). It is interesting future subject to analyze the effect of food additives on these alternative STAT3 signaling pathways.

In this study, we investigated food additives containing aldehyde structures, building on the growing evidence linking aldehydes to obesity. Notably, elevated tissue levels of 4-HNE, a well-known aldehyde compound, have been shown to contribute to obesity and insulin resistance (48), and have been associated with fat accumulation in both nematodes and mice through increased citrate levels (49). Moreover, we have previously reported 4-HNE involvement in leptin resistance (37), leading us to hypothesize that food additives containing aldehyde structures could also contribute to this outcome. Our results support this hypothesis, showing that certain food additives with aldehyde structures (#40, #42, #44) inhibit leptin-induced transcriptional activity in neuronal cells, suggesting they could contribute to leptin resistance and potentially promote obesity. This finding aligns with several other studies that have reported associations between food additives and obesity. Food additives are thought to contribute to immune-mediated metabolic dysregulation, increasing the risk of obesity and metabolic syndrome (50). Additionally, synthetic additives have been shown to interfere with endocrine function, insulin signaling, and adipocyte function, acting as chemical agents that induce obesity (51). Furthermore, artificial food colorings have been linked to behavioral changes in children through antinutritional mechanisms (52). While these findings highlight the potential role of food additives in the development of obesity, further research is needed to investigate the *in vivo* effects of #40, #42 and #44 food additives on leptin resistance and metabolic dysfunction.

For busy modern people, there is a demand for meals such as UPF that can be easily and quickly consumed. However, such UPF also contains many food additives (53, 54). Food additives are thought to play an important role in human nutritional intake, as they can improve appetite and Quality of Life (QOL) by improving the flavor and appearance (color). On the other hand, in this study, results obtained using cultured cells showed that some food additives have the potential to induce obesity. Although further verifications are required to analyze at the *in vivo* and *in vitro* levels, the correct use of food additives will be expected to lead to the development of healthy and delicious foods for nutritional intake.

This study is the first to demonstrate that food additives containing aldehyde structures may attenuate leptin action in neuronal cells, potentially leading to leptin resistance. We also explored the possible mechanisms of action underlying this effect. Our findings suggest that food additives containing three aldehyde structures #40, #42, #44 (2-pyrrolecarbaldehyde, o-anisaldehyde, piperylene) contribute to leptin resistance through a mechanism that could involve leptin-regulated STAT3 transcriptional activity. Considering the molecular mechanisms of the leptin resistance, additional studies are still needed to further elucidate the underlying mechanisms and to confirm the translational relevance of these findings. It is known that endogenous reactive aldehydes will affect DNA replication or mRNA transcription (55–58). In the present study, we found that several types of aldehyde containing food additives reduced STAT3 transcriptional activity. These results will evoke the hypothesis that aldehyde containing food additives may also affect DNA replication or mRNA transcription by binding with DNA or protein, including transcriptional coactivators (59).

While food additives are generally considered safe and non-toxic in small amounts, the long-term effects of chronic consumption on living organisms remain unclear. In this study, we examined the possibility of obesity as a risk of hidden side effect of food additives. In the present study, we observed that some food additives at 10–100 μM reduced leptin signaling in a cellular level. However, it is unknown whether the concentrations tested are realistically achievable through human dietary exposure. It would be possible that chronic administration of food additives over years or even decades may lead to accumulation in the body, leading to high concentrations. However, currently it is unknown whether the food additives will accumulate in human body. It is the limitation of the current experiments and future studies are required to understand these possibilities.

Our current results suggest that certain aldehyde-based food additives may pose a risk in the development of obesity. These findings highlight the need for further research into the metabolic effects of food additives and their relevance to human health. Also, given the broad role of STAT3 signaling in cellular regulation, proliferation, survival, inflammation, and tumor progression (60, 61), our results may extend beyond the metabolic disorders.

## Materials and methods

### Cells culture

The cells used were human neuroblastoma SHSY5Y-ObRb cells (62), which permanently express Ob-Rb leptin receptors in human neuroblastoma SHSY5Y cells (62). SHSY5Y neuroblastoma cell line was originally obtained from ATCC (American Type Culture Collection). SHSY5Y-ObRb cells were previously established cell line at our group, which stably express Ob-Rb leptin receptor in SHSY5Y neuroblastoma cell line (62). The SHSY5Y-ObRb cells were cultured at DMEM medium containing 10% FCS and 1% Penicillin–Streptomycin Solution (x 100; Wako, Japan).

### Reagents

We dissolved all aldehyde-containing compounds used in food additives to a concentration of 10 mM stock in DMSO (Fujifilm Wako Pure Chemical, Japan). In the experiments, the concentration was adjusted using DMEM (Fujifilm Wako Pure Chemical, Japan). Concentrations of the compounds used for treatments were 3, 10, 30 and 100 μM. For the ER stress inducing reagents we used tunicamycin (Tm; Fujifilm Wako Pure Chemical, Japan) and thapsigargin (Tg; Sigma) with the final concentration at 1 μg/mL and 0.1 μM, respectively.

We used following food additives in the present study: #1_2-Methyl-3-(3,4-methylenedioxyphenyl) propionaldehyde (Tokyo Chemical Industry, Japan), #2_(+−)-Citronellal (Tokyo Chemical Industry, Japan), #3_3-methylthiophene-2-carboxaldehyde (Tokyo Chemical Industry, Japan), #4_geranyl formate (Tokyo Chemical Industry, Japan), #5_Isopentyl formate (Tokyo Chemical Industry, Japan), #6_Citronellyl formate (Fujifilm Wako Pure Chemical, Japan), #7_α-amyl cinnamaldehyde (Tokyo Chemical Industry, Japan), #8_Acetaldehyde (Fujifilm Wako Pure Chemical, Japan), #9_p-methoxy benzaldehyde (Fujifilm Wako Pure Chemical, Japan), #12_Iso valeraldehyde (Tokyo Chemical Industry, Japan), #13_Isobutyl aldehyde (Tokyo Chemical Industry, Japan), #14_n-octanal (Tokyo Chemical Industry, Japan), #15_TRANS-Cinnamaldehyde (Nacalai tesque, Japan), #16_Decanal (Tokyo Chemical Industry, Japan), #17_Barrel aldehyde (Tokyo Chemical Industry, Japan), #18_Butyraldehyde (Tokyo Chemical Industry, Japan), #19_Propionaldehyde (Nacalai tesque, Japan), #20, Benzaldehyde (Nacalai tesque, Japan), #21_(−)-Peryl aldehyde (Tokyo Chemical Industry, Japan), #22_3-methyl-2-butenal (Tokyo Chemical Industry, Japan), #24, 3-ethoxy-4-hydroxybenzaldehyde (Tokyo Chemical Industry, Japan), #25_2,3-dimethoxybenzaldehyde (Tokyo Chemical Industry, Japan), #26_o-vanillin (Tokyo Chemical Industry, Japan), #28_Nonanal (Fujifilm Wako Pure Chemical, Japan), #29_3-Phenylpropionaldehyde (Tokyo Chemical Industry, Japan), #30_10-Undecenal (Tokyo Chemical Industry, Japan), #31_3-(4-Isopropylphenyl) iso butyl aldehyde (Tokyo Chemical Industry, Japan), #32_α-Hexyl cinnamaldehyde (Tokyo Chemical Industry, Japan), #33_50% phenylacetaldehyde diethyl phthalate solution (Fujifilm Wako Pure Chemical, Japan), #34_Cumin aldehyde (Tokyo Chemical Industry, Japan), #35_Dodecanal (Fujifilm Wako Pure Chemical, Japan), #36_3-Thiopehenecarboxaldehyde (Fujifilm Wako Pure Chemical, Japan), #37_ 5-Methylthiophene-2-carboxaldehyde (Tokyo Chemical Industry, Japan), #38, trans-2-Heptenal (Tokyo Chemical Industry, Japan), #40, 2-pyrrolecarbaldehyde (Fujifilm Wako Pure Chemical, Japan), #41_3-(2-Furyl) acrolein (Fujifilm Wako Pure Chemical, Japan), #42_o-anisaldehyde, #43, L-(+)-Arabinose (Fujifilm Wako Pure Chemical, Japan), #44, Piperonal (Fujifilm Wako Pure Chemical, Japan).

### Luciferase assay

The cells used were SHSY5Y neuroblastoma cells, which stably express ObRb leptin receptor and sis-inducible element (SIE) that drives transcription of the luciferase reporter gene luc2P (pGL4.47[luc2P/SIE/Hygro], Promega). Activated STAT3 induced by leptin interact with SIE, which drives luciferase reporter gene. The cells were seeded at 80,000 cells/well. Stimulation with the compounds were done by replacing the medium with reagent-containing medium, and 1 h later, leptin (0.01 μg/mL) was added. Luminscence measurements were performed 2 h after adding the leptin. The measurement was done according to the Nano-Glo Luciferase Assay procedure (Promega N1110) using the Infinite® 200 PRO (TECAN).

### Cell viability assay

The cells were seeded at 80,000 cells/well, with 200 μL in each transparent 96-wells. The medium was replaced with food additives containing aldehydes under the same conditions. Cell viability assay was performed using a WST-8 based cell cytotoxicity assay per as the manufacture’s protocol (Dojindo, Japan). One hour later, the absorbance at 450 nm was measured.

### Western blotting analysis

For Western blotting, cells were washed with ice-cold PBS and lysed in a buffer containing 1 M HEPES-HCl (pH 7.5) 0.4 mL 5 M NaCl 1.2 mL 0.5 M EDTA 80 μL 20% NP-40 2 mL MilliQ 36.32 mL for 20 min. The lysates were centrifuged at 15,000 rpm for 20 min at 4 °C, and the supernatants were collected. The sample were fractionated by SDS-PAGE, and transferred at 4 °C to nitrocellulose membranes. The membranes were incubated with anti-STAT3 (proteintech,10,253-2-AP,1:2000 dilution), anti-Phospho-STAT3 (Cell Signaling Technology,9,145,1:2000 dilution), anti-GAPDH (Fujifilm Wako Pure Chemical, Japan,01425524,1:2000 dilution), and anti-KDEL (Enzo, ADI-SPA-827,1:1000 dilution) antibodies, followed by secondary anti-rabbit or mouse antibody and detected by Dylight 800. KDEL antibody majorly detects GRP78 and GRP94, and the target protein was determined based on molecular weight. The intensity of the band was measured using ImageJ. The intensity of each protein was expressed as ratio of GRP78/GAPDH or P-STAT3/STAT3, respectively.

### Bioluminescence resonance energy transfer (BRET) analysis

The BRET assay was performed following the protocol described in a previous report (63). Briefly, leptin receptor fused with luciferase (OBR-2 K-Rluc, energy donor) and leptin receptor fused with YFP (OBR-2 K)-YFP, energy acceptor), were transfected into HEK293T cells using polyethyleneimine (PEI) and cultured for 24 h. The cells were then plated to a 96-well plate (PerkinElmer, Culture Plate™-96), and cultured for an additional 20 h. Food additives (1 h pretreatment, 100 μM) and leptin (10 nM, 30 min) were added. For the BRET assay, the plate was washed with PBS, and Coelenterazine h (Interchim, France) was added. Luminescence signals were detected in an Infinite 500, Tecan. Results were expressed as milliBRET units (mBRET units) = {(YFP/Rluc)–(YFP/Rluc of cells expressing the donor alone)} × 1,000.

### Immunocytochemistry

SHSY5Y-ObRb cells were cultured in a 3.5 cm^2^ dish with slide glasses. Then the cells were stimulated with various aldehydes (#40: 2-Pyrrolecarbaldehyde (Fujifilm Wako Pure Chemical, Japan), #42: o-Anisaldehyde (Tokyo Chemical Industry, Japan), and #44: Piperonal (Tokyo Chemical Industry, Japan); each at 100 μM) for 1 h, followed by leptin (Enzo, 0.01 μg/mL) for 15 min. After stimulation, cells were washed twice with TBS and fixed with methanol. Blocking was performed with 5% BSA at 37 °C for 1 h. Subsequently, cells were washed twice with TBS-T (0.1%) and incubated overnight at 4 °C with the anti-Phospho-STAT3 (Y705) antibody (rabbit, 1:100; Cell Signaling Technology, 9,145; Danvers, MA). Subsequently, secondary antibody treatment was done using Alexa 488 anti-rabbit antibody (rabbit, 1:2000; Invitrogen, MA). Nuclear staining was performed using Hoechst (346–07951; 1/1000 dilution, Dojindo, Japan). After staining, coverslips were mounted onto slide glasses using FluorSave™ Reagent (Millipore, MA) as the mounting reagent. Imaging was performed using a fluorescence microscope BZ-X810 (Keyence Corporation, Japan). To quantify immunoreactivity, all images were captured under identical acquisition settings across experimental groups. The cell counts of immunoreactivity-positive cells, as well as fluorescence intensity analysis, were automatically performed for each captured image using the hybrid cell count software BZ-X Analyzer (Keyence Corporation, Japan).

### Statistics

Results are expressed as the mean ± S. E. Statistical analysis was performed using the One-way ANOVA followed by Tukey–Kramer (Figures 2B–D, 3B, 5B, 6B) tests for the multiple comparison of the data. The data was obtained followed by a normal (Figures 2B–D, 3B, 6B) or non-normal (Figure 5B) distribution. Statistical analyses were performed using GraphPad Prism software (version 8, San Diego, CA, USA) and Microsoft Excel (Microsoft Corporation, Redmond, WA, USA).

## Data Availability

The original contributions presented in the study are included in the article, further inquiries can be directed to the corresponding author.
